# Gluten Free Diet for the Management of Non Celiac Diseases: The Two Sides of the Coin

**DOI:** 10.3390/healthcare8040400

**Published:** 2020-10-14

**Authors:** Diana Di Liberto, Daniela Carlisi, Antonella D’Anneo, Sonia Emanuele, Michela Giuliano, Anna De Blasio, Giuseppe Calvaruso, Marianna Lauricella

**Affiliations:** 1 Department of Biomedicine, Neurosciences and Advanced Diagnostics (BIND), CLADIBIOR, University of Palermo, 90127 Palermo, Italy; 2 Department of Biomedicine, Neurosciences and Advanced Diagnostics (BIND), Institute of Biochemistry, University of Palermo, 90127 Palermo, Italy; daniela.carlisi@unipa.it (D.C.); sonia.emanuele@unipa.it (S.E.); marianna.lauricella@unipa.it (M.L.); 3 Department of Biological, Chemical and Pharmaceutical Sciences and Technologies (STEBICEF), Laboratory of Biochemistry, University of Palermo, 90127 Palermo, Italy; michela.giuliano@unipa.it (M.G.); anna.deblasio@unipa.it (A.D.B.); giuseppe.calvaruso@unipa.it (G.C.)

**Keywords:** gluten, non celiac disease, gluten-free diet

## Abstract

A lifelong adherence to a gluten-free (GF) diet is currently the only treatment for Celiac disease (CD), an autoimmune disorder that arises after gluten ingestion in individuals who are genetically predisposed. The gluten intake exerts toxic effects through several pathways involving gut barrier integrity, intestinal microbiota composition and immune system stimulation. However, despite the great benefit of GF diet for CD patients, its use has been debated. Indeed, individuals who adopt this diet regime may be at risk of nutrient deficiencies. Emerging evidence supports a beneficial effect of a GF diet also for other pathological conditions, including gluten-related disorders (GRD) often associated to CD, such as Non celiac gluten sensitivity (NCGS) and Dermatitis Herpetiforme (DH) as well as Irritable bowel syndrome (IBS) and Diabetes. This suggests a pathogenic role of gluten in these conditions. Despite the growing popularity of GF diet among consumers, to date, there are limited evidences supporting its use for the management of non-celiac diseases. Therefore, in this review, we discuss whether the GF diet could really improve the general quality of life of patients with GRD and non-GRD conditions, keeping in mind its sensorial limitations and nutritional inadequacies. In addition, we discuss the current motivations, leading to the use of a GF diet, despite the inferior quality of GF products respect to those containing gluten.

## 1. Introduction

Gluten is a complex molecule present in several grains, including wheat, rye an barley [1], consisting of glutenin polymers and gliadin monomers. Glutenin can be divided into proteins with high and low molecular weight, while gliadin belongs to a large family of proteins consisting of α,-β-,γ- and ω-types [2]. Both glutenin and gliadin contain high percentage of prolines (20%) and glutamines (40%) protecting them from complete degradation in the gastrointestinal tract and resulting in their incomplete digestion [2]. Among gliadin peptides, the most characterized are the 57–89 peptide (33-mer) α-gliadin fragment [3], the cytotoxic peptide, the gut permeating peptides and the IL-8 releasing peptide [4].These undigested peptides have been demonstrated to play biological activities in gastrointestinal tracts, including increased gut permeability and cytotoxic and immunomodulatory effects [5].

Gluten ingestion is responsible for the development of Celiac Disease (CD), an autoimmune enteropathy activated in the lamina propria of the gut of genetically predisposed individuals [6] by gliadin peptides and resulting in the recruitment of infiltrating T lymphocytes producing Interferon-gamma (IFN-γ) and Interleukin-15 (IL-15). The pathogenesis of CD is related to both genetic and environmental factors. Among genetic factors, major histocompatibility complex (MHC) class II, HLA DQ2 and DQ8, confer the greatest susceptibility to the disease, with a reported gene dosage effect according to which homozygous allotypes are associated to an increased risk [7,8]. In addition, among environmental factors, gastrointestinal dysbiosis seems to be associated to CD onset, consisting of an increased number of *Proteobacteria* and *Bacteroidetes* and a reduced number of *Firmicutes,* especially in the active phase of the disease [9].

Clinical manifestations of CD are mainly gastrointestinal symptoms, such as intermittent or chronic diarrhea, bloating and abdominal distension, colitis, flatulence, abdominal cramps, in association with malabsorption and consequent iron deficiency and weight loss. Extraintestinal symptoms are chronic fatigue, foggy mind, aphtous stomatitis, reduced bone density, growth retardation in children and short stature [10].

CD diagnosis is a multi-step process. First, patients are screened for serum IgA anti-tissue transglutaminase, if they have detectable IgA level [11], otherwise serum IgG anti-tissue transglutaminases are preferred. In addition, they can be screened for serum IgG anti-deamidated gliadin, an alternative test with an increased sensitivity and specificity [1], and anti-endomysium IgA, to confirm borderline results. Finally, a full-blown diagnosis requires small intestinal biopsy showing villous atrophy, an increased number of intraepithelial lymphocytes and elongated crypts [10].

Experimental evidences showed that gliadin peptides are capable to trigger an adaptive immune response in CD patients, as demonstrated by the higher production of pro-inflammatory Th1/Th17 derived cytokines from intestinal biopsies of active CD patients when stimulated in vitro with gliadin [12]. In addition, peripheral blood mononuclear cells of CD patients produce Interleukin-1 β (IL-1β) and Interleukin-18 (IL-18) following their stimulation with gliadin [13]. Interleukin-15 (IL-15) is also up-regulated in the epithelium and the lamina propria of CD patients in the active phase [14]. To explain the toxic effect of gluten, it was hypothesized that, after gluten oral introduction, partially digested gliadin peptides, interacting with the small intestinal mucosa, may first activate an innate immune response. This is evidenced by the production of IL-8 by epithelial and lamina propria dendritic cells. IL-8, in turn, recruits neutrophils in situ amplifying the inflammatory process, whereas IL-15 induces enterocytes apoptosis [15]. Then, gliadin peptides, through the interaction with CXCR3 receptors present on the apical side of the epithelium, seem to trigger the release of zonulin, the regulator of the intestinal tight junctions, leading to an increase in intestinal permeability and consequent antigen trafficking that may cause autoimmune disorders [16,17]. As a consequence of the increased intestinal permeability, gliadin peptides may translocate into the lamina propria where they undergo to deamidation by transglutaminase 2 [18]. This event has been shown to favor gliadin interaction with macrophages and dendritic cells of the submucosa [19], triggering an adaptive immune response with the production of increased pro-inflammatory cytokines such as IFN-γ, tumor necrosis factor-alpha (TNF-α) and IL-17 [19], which damage the intestinal mucosa compromising its permeability [20].

Currently, the only treatment of CD is the complete elimination of gluten from diet, which leads to symptoms disappearance. However, about 7–30% of CD patients do not fully improve after adopting a gluten-free (GF) diet. Therefore, alternative CD therapies are required, such as genetically modified gluten, inhibitors of zonulin and supplementary probiotics [21,22,23,24].

Notably, gluten elimination studies performed in non celiac individuals showed that a GF diet exerts beneficial effects in preventing and/or reducing other conditions including both Gluten-related disorders (GRD), such as Non-celiac gluten Sensitivity (NCGS) and Dermatitis herpetiformis (DH), as well as non GRD, including Irritable Bowel Syndrome (IBS) and Diabetes. Thus, although the pathogenetic mechanisms are quite different, gluten seems to favour the onset/developmental of these diseases acting with similar mechanisms.

In this review, we critically discuss the potential beneficial effects of a GF diet for the management of NCGS, DH, IBS and Diabetes, highlighting, when possible, the mechanisms by which gluten may exert its pathogenetic effects.2. Gluten-Free Diet

The Gluten-free (GF) diet was introduced for the treatment for CD since 1941, as reported by the paediatrician Willem Karl Dicke [25], and is still in use for the management of this disease. Currently, it is also recommended for patients with other conditions including NCGS, DH, IBS and diabetes.

Today, a lot of research focuses on the understanding of knowledge, current trends and nutritional adequacy surrounding the GF diet and its effectiveness in GRD, diabetes, autoimmune disorders as well as in losing weight [26]. The global forecasts of GF products sales indicate an increase of 10.4% between 2015 and 2020 [27]. The European Parliament Regulation (EU) No 609/2013, in 2013, established the rules on composition and labelling of GF products. This is important to protect individuals with gluten intolerance from consuming products that could lead to worsening of symptoms and to correctly inform them on the differences, existing between naturally GF foods and foods that become GF after different processes [28]. Indeed, GF foods can be distinguished into those naturally GF, such as rice, corn, potatoes, some grains, seeds and legumes, and those that become GF after purification, a process that alters their macro-and micro-nutrient composition, as well as their nutritional values [28].

Gluten contained in wheat flours owns the unique ability to form aggregates, which are important for some products such as, bread, pasta and pretzels, whose production process requires a cohesive dough. Gluten is composed mostly of gliadin, the 70% ethanol-soluble protein fraction of wheat flour, and by glutenin and both contribute to the cohesiveness and to the elasticity of the dough [29].

Notably, wheat is not only a source of protein but also a food rich in micronutrients, including folate, iron, B vitamins (thiamine, riboflavin and niacin) and fibers. Therefore, a disadvantage in using a GF diet my result by the fact that often GF products have a lower amount of these components when compared to their gluten containing equivalents [30,31,32]. In particular, fibers are lost because they are mostly contained in the outer layer of grain which is eliminated during the refining process. Gluten replacing ingredients are starches, hydrocolloids, gluten-free flours of cereals/pseudocereals, proteins, enzymes and emulsifiers. These components are used in combination to improve rheological features of GF products, often leading to their substantial price increase [33].

Starch is the major component of wheat flour (80%), acting as an inert filler during the first steps of the bread making since gelatinizes after cooking. Alternatives to starch, cassava, tapioca, potato, corn and rice can be considered the “leading actor” in the structure setting of the bread, [34,35]. Recently, a synthesized GF wheat flour has been tested together with rice flour and corn starch for producing GF products: Wheat starch bread tasted better, also having an improved loaf volume when compared to corn starch bread [36,37]. Another additive often used to improve the quality of GF products is dietary fibers, such as β-glucan, inulin, carob or bamboo fiber, polydextrose and oligofructose [38,39,40]. These ingredients not only compensate the nutritional loss of fibers, but also increase viscosity and gel-forming ability, due to their water-binding capacity.

Notably, several experimental evidences showed that some GF products are foods richer in carbohydrates and lipids than their gluten containing equivalents [41], as for GF bread with a higher fat content and glycemic index and with a lower amount of proteins. The glycemic index of GF products changes according to the type and quality of contained ingredients and to the procedures used to obtain them [42]. In addition, GF products have generally lower amounts of folate, iron, riboflavin, niacin and thiamine [43] and researchers in this field work to overcome these limits without altering their taste [44]. Therefore, as reported by Barone and colleagues [45], CD patients compared to healthy individuals, assume higher amounts of sugar and fat and lower quantities of fibers when they are on a GF diet, but this may depend on their different habits. Indeed, when compared to the total population, CD patients consume the same quantity of cereal-based products but larger quantities of biscuits and crackers, may be due to the reduced tastiness and availability of the other GF products, such as bread [46,47]. Moreover, low plasma levels of calcium, iron, selenium, magnesium, zinc, niacin, thiamine, riboflavin and Vitamins A and D, were found in CD patients after a GF diet period [32,48]. This could be due to the impaired absorption characterizing CD patients. However, it has been shown that after 10 years of GF diet the higher homocysteine plasma levels detected in CD patients, when compared to the general population, reflected the deeper deficiencies in folate, vitamins B6 and B12 not associated to the intestinal mucosa damage [49]. In addition, nutritional GF diet-associated deficiencies have been shown to be attributable to different conditions such as dietary education, gender, health awareness and lifestyle factors that may influence food choices and scientific results on the effect of GF diet [50].

In consideration of nutritional deficiencies resulting from a GF diet, nowadays research focuses on trying to find wheat varieties having a lower or absent toxicity suitable for the treatment and prevention of CD, such as diploid wheat species who are not able to activate T cell response in the gut of CD patients [51]. The reduced or absent toxicity of diploid and ancient wheat species, such as *Triticum monococcum* ssp. *Monococcum* wheat, was demonstrated by in vitro cellular assays, confirming their potential management in new dietary strategies for CD patients [52]. However, little is known about the low toxicity of *Monococcus* wheat for CD patients, even if it is believed that not all the wheat varieties might be equally toxic. Gianfrani et al. [53] evaluated the toxicity of gliadin from two monococcum lines, Monlis and ID3311, after an in vitro simulation of human digestion with pepsin and trypsin, and suggested that both lines activate a T cell response. However, ID3311, being unable to activate an innate immune response, seems to be less pathogenetic and less effective in the induction of CD [53]. Moreover, the same group of CD patients showed that gliadin proteins of Monlis and ID3311 are less toxic following the in vitro simulation of human digestion. This could be due to the different protein content between the two monococcum lines, Monlis and ID3311, and the common wheat *Triticum aestivum.* Several monococcum peptides, such as immunodominant T cell epitopes, are degraded during gastrointestinal digestion unlike peptides from *Triticum aestivum* that instead survived during this process [54]. Therefore, although different clinical trials have recently highlighted the monococcum toxicity for CD patients, based on serological and also histological signs, it seems a safe cereal for CD prevention or for patients affected by NCGS [55].

Thus, the use of a GF diet may imply disadvantages related to nutritional deficiencies and high carbohydrate and fat contents. Such a condition could be probably improved if manufacturers and health professional collaborated to continuously adjust and improve the formulations and the processing techniques used in GF manufacturing, in order to ensure the consumer a diet that is as balanced and tastier as possible.

## 2. GF Diet and Non Celiac Gluten Sensitivity

Non celiac gluten sensitivity (NGCS) is a condition associated with a wide range of both gastrointestinal and extra-gastrointestinal symptoms improving after gluten removal and reappearing after gluten ingestion. These symptoms may include bloating, abdominal discomfort and pain, altered bowel habits, flatulence, rash, fatigue, headaches, mental disturbances, irritability, depression, bone and joint pain, and attention deficit disorder. There is large overlap between Irritable Bowel Syndrome (IBS), a common condition affecting the digestive system and causing symptoms like stomach cramps, bloating, diarrhoea and constipation, and NCGS, so that the medical literature has not yet explored the effects of a GF diet or other dietary manipulations in each of them [56].

The mechanisms by which gluten induces symptoms in NCGS are largely unknown, but the difference between NCGS and CD appears clear. In contrast to celiac disease, NCGS patients, by definition, do not have serological celiac disease–associated antibodies, they could be HLA-DQ2/8–negative and they also should not have flattened the villi in their small intestine. Whereas, CD leads to increased small intestinal permeability and activation of the adaptive immune response, literature data reported that NCGS patients have normal intestinal permeability and activation of the innate immune response without activation of the adaptive immune response [57,58,59]. However, a recent paper shows that also the adaptive immunity may be involved in the pathogenesis of NCGS, since the production of TNF-α by T helper and cytotoxic T cells, as well as of IL-17 by T helper cells, is higher in the rectal tissue of NCGS patients than in healthy individuals [60].

All the patients with IBS-like symptoms, and diagnosed as not CD patients, are classified as NCGS patients if they improve after gluten removal from diet and get worse after gluten reintroduction. Therefore, albeit the exact pathogenic mechanism of gluten is still unknown for NCGS, the benefits of gluten removal from diet is evident for these patients, thus, suggesting the use of a GF diet despite the disadvantages that can arise from this dietary regimen.

Interestingly, it is well known that, beyond gluten proteins, other ingredients contained in wheat are able to trigger symptom appearance in patients affected by NCGS. Among these there are the Amylase-trypsin inhibitors, that activate the innate immune system on myeloid cells via Toll-like receptors 4 (TLR4), Wheat germ agglutinin, a lectin that protects wheat from insects, yeast and bacteria, and fermentable oligosaccharides, disaccharides, monosaccharides and polyols (FODMAPs) [56,61]. For this reason, some researchers proposed the term non-celiac wheat sensitivity (NCWS) instead of NCGS [62], as a more inclusive term, taking into account of the other substances present in the wheat, besides gluten, eliciting the activation of the immune system and consequently also the appearance of symptoms [62,63]. Confirming this, it was reported that some patients with IBS-like symptoms benefited from a low-FODMAPs diet while other patients on a GF diet responded to a simultaneous restriction of FODMAPs [64].

Recently, fructan alone versus gluten versus placebo was evaluated in a randomized, double-blind, placebo-controlled, crossover study with self-reported NCGS patients [65]. This study revealed that IBS symptoms and bloating got worse following fructan ingestion when compared to gluten [65], but not following fructan ingestion versus placebo or gluten ingestion versus placebo.

Other studies showed a significant increase in symptom scores with a gluten challenge although only 16% of NCGS patients showed gluten-specific symptoms with a 40% of patients having a nocebo response [66,67,68]. In addition, the effects of gluten on mental state were evaluated by Peters and colleagues in patients with a self-reported NCGS recruited from the trial by Biesiekierski and colleagues [69,70]. Patients were included in the study only if they met Rome III criteria for IBS, and showed an improvement in symptoms on a GF diet for at least 6 weeks. CD patients were excluded from this study.

A validated 80-question survey (State-Trait Personality Inventory) reported that gluten ingestion was associated with higher depression scores compared to placebo while anxiety, anger, or curiosity scores were similar between the two groups.

Thus, although these studies support the existence of NCGS as an entity on its own, it appears that NCGS patients are included among patients with IBS-like symptoms. This confirms the existence of an overlap between IBS and gluten-related disorders (CD, NCGS, DH) and suggests that GF diet can benefit both gluten sensitive patients who report gluten-related symptoms and IBS patients who are also gluten or wheat sensitive.

## 3. GF Diet and Dermatitis Herpetiforme

Dermatitis Herpetiforme (DH), first described by Louis Adolphus Duhring in 1884 [71], is a chronic, autoimmune papulovesicular disorder associated to CD and NCGS with a male predominance of 1.44:1 to 2:1 that generally arises in the fourth decade of life, although it has been reported in younger and older patients [72]. Patients with DH are also characterized by an increase in intestinal permeability and zonulin up-regulation [73,74], which suggests a common pathogenetic mechanism in both CD and DH disorders. Gluten seems to be responsible for the intestinal abnormalities, characterizing both disease, as well as for the cutaneous immunoreactant deposition, with consequent skin disease activity, in DH [73]. It is postulated that the altered intestinal permeability correlated with gluten pepides may facilitate the entry of gluten or its peptides into the lamina propria where it causes a cascade of immunological events [74].

DH is characterized by subepidermal bullae detected by hematoxylin and eosin staining and granular deposits of immunoglobulin A (IgA) in the dermal papillae detected by direct immunofluorescence method. Biopsy is required for a definitive diagnosis of DH, although antibodies to tissue and epidermal transglutaminase are detectable in the serum of patients.

In addition to being associated with more than 90% of CD cases, DH can be also found in other autoimmune conditions, such as Hashimoto’s thyroiditis and other thyroid dysfunctions [75]. It has also been reported in patients with rheumatoid arthritis, Sjögren’s syndrome, lupus erythematosus, sarcoidosis, type I diabetes, vitiligo, primary biliary cirrhosis, pernicious anemia and Addison’s disease [76].

Patients with DH, as well as patients with the other autoimmune disease listed above, have a higher incidence of non-Hodgkin’s lymphomas compared to the general population and the risk of lymphoma seems to be reduced with a strict adherence to GF diet [77]. In fact, GF diet is the first-line therapeutic approach improving both cutaneous and intestinal manifestations of disease, while dapsone and sulfones target the skin lesions only. For a good prognosis, the initial treatment often requires a combined therapy with GF diet and dapsone, usually progressing to dose reduction after a few months. A close monitoring of DH patients is recommended during therapy by a medical team consisting of dermatologists, dieticians, gastroenterologists and rheumatologists, given the high dapsone toxicity and the association of other autoimmune disorders to DH.

## 4. GF Diet and Irritable Bowel Syndrome

Irritable bowel syndrome is a gastrointestinal disorder, affecting 9–23% of the world population (World Gastroenterology Organization, 2009) [78] and characterized by altered bowel functions associated with abdominal discomfort or pain, without detectable structural and biochemical changes [79].

IBS can negatively affect quality of life and is the most commonly diagnosed gastrointestinal condition. The pathogenesis is poorly understood and many factors are involved, such as altered gastrointestinal motility, visceral hypersensitivity, post infectious reactivity, brain-gut interactions, bacterial overgrowth, alteration in faecal microflora, food sensitivity, carbohydrate malabsorption and intestinal inflammation [80]. Symptoms that patients frequently complain about are predominantly abdominal pain or discomfort, bloating, diarrhoea, and constipation. Some symptoms are also extra gastrointestinal, like fatigue, commonly recovered among IBS patients [80]. IBS is diagnosed on the basis of the presence of characteristic symptoms and the exclusion of other organic diseases. Treatment of IBS patients is a personalized approach consisting of different aspects in addition to the medical one, such as those concerning diet, lifestyle, and behaviour [80].

Diet has a critical role in IBS patients with up to 84% reporting food-related symptoms [81,82,83], especially after ingestion of FODMAPs and fats containing foods [83].

Different mechanisms have been proposed by which food may lead to symptoms appearance in IBS patients. These are divided into primary effects, like pro-, pre-, and post-biotic, osmotic, mechanical, chemical and neuroendocrine, and secondary effects, including fermentation by-products, alterations in intraluminal pH and consequent effects on the microbiome [84]. Traditional dietary advices, including healthy eating and lifestyle management, are contained in the guidelines provided by the National Institute for Health and Care Excellence (NICE) and the British Dietetic Association (BDA) and they include adequate fluid and fiber intake, regular meals, assessment of alcohol and caffeine intake, decreasing fat and spices intake. A reduction of alcohol and caffeine consumption is recommended, given the effects of the first on gastrointestinal motility and permeability and being a colonic stimulant the second, [85,86].

Also, fatty foods are known to exacerbate symptoms in IBS patients resulting in an increased gastrocolonic response [81,83,87].

Moreover, a low FODMAPs diet is currently suggested as second-line therapy for IBS [85], although some suggest it as potentially first-line therapy [88]. FODMAPs are short-chain carbohydrates, osmotically active and poorly absorbed, which increase small intestine water content and transit. They are rapidly fermented in the colon, leading to intestinal gas production and distention [89].

Probably, colonic hypersensitivity is the pathophysiological mechanism by which FODMAPs intake generates symptoms in individuals with IBS [90].

The low FODMAPs diet should be implemented under dietitian monitoring, with a first elimination phase for 4–8 weeks followed by a reintroduction phase where FODMAPs are gradually reintroduced to induce tolerance in IBS individuals. In the long term, an ‘adapted’ low FODMAP diet appears to be nutritionally adequate, although these data are limited to only one study [91].

Over the last decade, research has also focused on the effect of gluten addition and removal from IBS patients diet [92]. The use of a GF diet, not only for CD treatment, have become a global phenomenon with up to 10% of the population self-reporting NCGS with gluten ingestion induced IBS-like symptoms [93].

Biesiekierski and colleagues showed that, in 34 IBS patients who did not have CD, gluten intake was associated with both gastrointestinal and no gastrointestinal symptoms [94]. In this study, patients randomly received gluten or placebo for six weeks and they were followed up. Gluten-ingesting patients had, within one week, more severe IBS-like symptoms, such as pain, bloating, satisfaction with stool consistency, and tiredness in the absence of significant changes in faecal lactoferrin, celiac antibodies and C-reactive protein levels or intestinal permeability.

Another trial reported by Vazquez-Roque and colleagues highlighted the effects of a 4 week GFD, compared to a gluten-containing diet (GCD), on daily bowel functions and transit, mucosal permeability and cytokine production in 23 diarrhoea-predominant IBS (IBS-D) patients diagnosed by Rome II criteria and without CD [95].

Patients on a GF diet had less peristalsis associated to a reduced intestinal permeability when compared to patients on a GCD, even if the adverse effects of gluten were higher in HLA-DQ2/8–positive patients, suggesting an adaptive immune response involvement in the gut inflammation and permeability increase triggered by gluten ingestion. Finally, Aziz and colleagues reported the results of a study performed with 41 IBS-D patients on a six weeks dietitian-led GF diet [96] both HLA-DQ2/8–positive (20 patients) and HLA-DQ2/8–negative (21 patients). After six weeks, GF diet treated patients (71%) referred an improvement with a decrease in IBS Symptom Severity Score of at least 50 points, remaining on a GF diet 18 month after the end of the study.

Therefore, while several studies reported the benefit of a GF diet in IBS, the mechanisms by which improvements occur are not yet fully understood. It is well-known that gluten can increase the intestinal permeability in IBS patients eating gluten-containing products, reducing the expression of tight junction proteins in the colonic mucosa. Moreover, in addition to gluten, other wheat components can also trigger symptoms IBS-like, including ATI and FODMAPs. Some pivotal studies suggest that the benefits of the GF diet may be due to the related effect of the reduced intake of FODMAPs, such as fructan, rather than of gluten per se [65,70]. In this regard, it could be speculated that the term ‘GF diet’ for IBS consists in the reduction of fructan intake, which some may consider as a ‘FODMAP-gentle’ approach [97].

## 5. GF Diet and Diabetes

Type 1 Diabetes (T1D) is an autoimmune disease characterized by a deficit or absence of insulin resulting from T cell-mediated destruction of β-cells of the pancreas [98]. The reduced production of insulin is responsible for the presence of high blood sugar level. In addition to hypoinsulinaemia and hyperglycaemia other classic symptoms of T1D include polyuria, polydipsia, weight loss and fatigue, which, if untreated, can lead to a coma and ultimately to death. Furthermore, T1D patients are characterized by the presence in serum of auto-antibodies against β-cell auto-antigens. Islet cell antibodies (ICA) were the first auto-antibodies identified in T1D patients [99]. In addition to ICA, other auto-antibodies associated with T1D are auto-antibodies to insulin (IAA) [100], glutamic acid decarboxylase (GADA) [101], and protein tyrosine phosphatase like protein (IA2) [102]. The highest incidence rate of T1D is found in adolescents, although it is widely accepted that adults can also develop the disease. T1D is a multifactorial disease where both genetic and environmental factors contribute to the pathogenesis. In genetically predisposed individuals, multiple and different environmental factors trigger an autoimmune response that causes the destruction of pancreatic β-cells. Several genome screens, in combination with family-based association studies, have shown that the most important genes responsible for the development of diabetes are those related to the Human Leukocyte Antigen (HLA), the locus of the genes that encode proteins, which facilitate the presentation of antigens to T lymphocytes. HLA is a group of polymorphic genes consisting of 30 units, located in humans on the short arm of chromosome 6. Approximately 50% of the genetic risk of T1D is correlated with the HLA class II region [103] and the presence of haplotypes HLA-DR3-DQ2 and HLA-DR4-DQ8 is the strongest determinants of diabetes susceptibility [104]. Other genetic polymorphisms that have also been associated with T1D development include those for the Insulin gene (INS) [105], IL-2 receptor α [106], Cytotoxic T lymphocyte antigen (CTLA4) [107], Protein tyrosine phosphatase non-receptor 22 (PTPN22) [108] and Intercellular adhesion molecule 1 (ICAM1) [109]. In addition to genetic components, many environmental factors have been associated with increased susceptibility to T1D, although, to date, none have been confirmed as a clear causative agent of T1D. The main environmental factors include viruses [110], vaccines, toxins and nutrients [111]. T1D patients have a high risk to develop other autoimmune disorders, such as Hashimoto syndrome and Celiac Disease [98].

At least 10% of patients with T1D develop Celiac disease at some point in their lives [112]. The comorbidity of T1D with CD has been attributed to an overlap in the genetic susceptibility to both diseases conferred by the HLA-DR3-DQ2, which is present in over 90% of individuals with celiac disease and 55% of those with T1D [113]. Interestingly, T1D rarely develops after diagnosed CD, indicating a protective role of GF diet for diabetes [114]. Many evidences strongly indicate that gluten is involved in the pathogenesis of T1D [115]. Notably, studies performed in non-obese diabetic (NOD) mice showed that a GF diet exerts a protective effect against the development of T1D [116]. Interestingly, a reduction of autoimmune diabetes development in NOD mice offspring occurs if a gluten-free diet is administered during pregnancy [117]. A beneficial effect of a GF diet has been also reported for human T1D patients. Notably, Sildorf et al. reported that following a GF diet a child with T1D remains without the need for exogenous insulin after 20 months [118]. Interestingly, a GF diet seems to be most effective when applied in utero, and the timing of the introduction of gluten seems to be critical. In fact, maternal ingestion of low versus high amounts of gluten during pregnancy has been shown to reduce by two fold the risk of T1D in their offspring [119]. In addition, it has been shown that taking gluten before three months increases the risk of developing T1D compared to receive only breast milk during this period [120]. However, there are only few studies in human T1D patients and scientists need to carry out further research.

Although, data reported in literature suggest that gluten may plays a role in the onset and development of T1D, the exact mechanism of its action is still unknown.

Change in intestinal microflora and gut permeability alteration may contribute to gluten-induced T1D development. The presence of gluten in the diet has been shown to alter microflora composition in the gut favouring the presence of *Bacteroides* species over other species such as *Bifidobacterium* and *Lactobacillus* [121]. This altered microflora composition is responsible for activation of zonulin, a molecule which increases gut permeability through the disassembly of thighs junctions [16]. Interestingly, T1D patients have shown an increased intestinal permeability and serum zonulin levels [122], as well as a decrease in bacteria that maintain the intestinal permeability [123]. Changes in microbiome composition seems to favour the development of T1D by either altering intestinal permeability or modifying immune system regulation [123]. Moreover, gluten also contributes to formation of antibodies and pro-inflammatory cytokines that cause inflammation [18]. Notably, upon stimulation with wheat proteins or their components, patients with T1D showed an increased proliferative T cell response and their PBMC produced significantly more pro-inflammatory cytokines, compared to control subjects [124,125,126,127]. Furthermore, alterations in gut permeability may favour the entry of gliadin peptides in association with other toxic molecules as LPS into the bloodstream of diabetes patients and contribute to β-cells damage. Notably, studies performed in mice showed that gluten peptides cross the intestinal barrier and accumulate in different tissues including pancreas [128]. Furthermore, gliadin fragments have been detected in breast milk, leading to hypothesize that gliadin cross the intestinal barrier also in humans [129]. Gluten peptides, which cross the intestinal barrier, may directly affect pancreatic β-cells. In both insulinoma INS-1E cells and in isolated rat islets, gliadin has been shown to increase insulin secretion by closing ATP-dependent K-channels [130]. This event may lead to β-cell hyperactivity and dysfunction, thus contributing to diabetes development. Moreover, it has been reported an effect of gluten in the stimulation of immune cells in the pancreatic lymph nodes of healthy mice [131]. This may contribute to local inflammation and β-cell stress, which could promote T1D development and may help to explain the beneficial effects of a GF diet in animal model of T1D.

Interestingly, several studies suggest that GF diet can also reduce the risk of type 2 diabetes (T2D). T2D is the most known and most frequent type of diabetes, which typically affects subjects of mature age. T2D develops in individuals who becomes resistant to insulin or are unable to produce enough insulin as a result of β-cells dysfunction [132]. As for as T1D, genetics and environmental factors are important disease determinants in T2D. Over 40 genes have been associated with T2D including peroxisome proliferator activated receptor gamma (PPARγ), ATP binding cassette subfamily C member 8 (ABCC8), and potassium voltage-gated channel subfamily J member 11 (KCNJ11). Among the environmental factors overweight and obesity, resulting from an unbalance between excess intake of food and insufficient physical activity, play a causal role in the pathogenesis of T2D [133]. Obese subjects as well as T2D patients have changes in the intestinal microbiota with an abundance of bacteria with an increased capacity to harvest energy and increase fat stores [134]. Moreover a reduced abundance of bacteria capable of producing butyrate [118], a short free fatty acid which decreases intestinal permeability [48], contributes to gut permeability alteration in T2D patients. This event in turn may favour the passage of LPS and gram-negative bacteria from the intestine to the adipose tissue and may contribute to low-grade inflammation, insulin resistance, β-cell dysfunction, and, thus, T2D. Considering that, as above reported, gluten seems to both increase the intestinal permeability and alter intestinal microbiota, the intake of gluten could therefore contribute to T2D by these mechanisms. Notably, studies in mouse models of T2D indicate that a GF diet alleviates the disease by improving the intestinal barrier function and contributes to a healthier microbiota. Moreover, it has been reported that stimulation of TLR4 receptor has been associated with insulin resistance and β-cell dysfunction in T2D [135]. This is relevant because gliadin has been demonstrated to induce insulin resistance and β-cell dysfunction through activation of TLR4. Moreover, the ability of gliadin of increasing insulin secretion in INS-1E cells as well as stimulating immune system cells may contribute to β-cell dysfunction in T2D patients. These data suggest that a GF diet may alleviate insulin resistance as well as β-cell stress and dysfunction in T2D.

Notably, if on the one hand gluten itself may contribute to the onset and development of T1D and T2D, on the other many food containing gluten, such as white bread, pasta and cakes, also contain sugars and carbohydrates, which can increase blood sugar levels. This may represent another advantage for the GF diet use in diabetic patients. However, some GF foods contain higher levels of carbohydrate and fat than foods that contain gluten and the fiber content may be lower. Therefore, when diabetic patients adopt a gluten free diet, they should choose the healthful foods and include nutritious high fiber in their meal planning.

## 6. Conclusions

The intake of gluten containing foods has been shown to cause toxic effects and contribute to the development not only of CD and GRD, but also of other diseases not strictly related to gluten intolerance, such as IBS and Diabetes. Although, the pathogenic mechanisms are different, the basis of gluten toxicity is the ability of gluten-derived peptides to induce intestinal permeability alteration, change in microbiota composition, as well as immune system stimulation (Table 1). Several clinical studies show how a GF diet brings benefits to individuals suffering from GRD, IBS, DH and diabetes, suggesting their use. However, the adherence to a GF diet, strictly necessary for celiac subjects, determines nutritional deficiencies as well as the risk of an excessive intake of fats and carbohydrates. Therefore, it is necessary to integrate micronutrients and fibers, which are lacking in gluten free foods, as well as follow controlled dietary regimes. In this regard, it is very important to adjust and improve the formulations of GF products, in order to ensure the consumer a diet that is as balanced and tastier as possible.

## Figures and Tables

**Table 1:** Different pathological conditions associated with Gluten intake.

	Celiac Disease (CD)	Non Celiac Gluten Sensitivity (NCGS)	Dermatitis Herpetiforme (DH)	Irritable Bowel Syndrome (IBS)	Type 1 Diabetes (T1D)	Type 2 Diabetes (T2D)
**Worldwide incidence**	0.7–1%	6%	0.01%	9–23%	2–5%	8.5%
**Correlation with HLA genes**	Restricted to HLA−DQ2/DQ8 [7,8]	Not restricted to HLA−DQ2/DQ8 [56]	Restricted to HLA−DQ2/DQ8 Associated to HLA−A1, −B8, DR3, −DQ2 [72]	Not restricted to HLA−DQ2/DQ8 [78,79]	Restricted to DRB1 * 03/DQB1 * 02:01/ B1 * 04/DQB1 * 03:02 [103,104]	Not associated to HLA−DQ2/DQ8 [132]
**Symptoms**	Diarrhea, bloating and abdominal distension, colitis, flatulence, abdominal cramps, malabsorption, and weight loss, chronic fatigue, foggy mind, aphtous stomatitis, reduced bone density and growth retardation [10]	Bloating, abdominal discomfort and pain, altered bowel habits, flatulence, rash, fatigue, headache, mental disturbances, irritability, depression, bone and joint pain, and attention deficit disorder [56]	Intestinal abnormalities and sub-epidermal bullae [71,72]	Abdominal pain or discomfort, bloating, diarrhoea, constipation and chronic fatigue [79,80,81]	Excessive hunger or thirst, weight loss, frequent urination, extreme fatigue, nausea, vomiting, or abdominal pains, visual impairment [98]	Excessive hunger or thirst, frequent urination, extreme fatigue, neuropathy and visual impairment [132]
**Diagnosis**	Small intestinal biopsy showing villous atrophy supported by serological tests (IgA anti-tissue transglutaminase, IgG anti-tissue transglutaminases, IgG anti-deaminated gliadin, IgA anti- endomysium) [10,11]	Small intestinal biopsy not showing villous atrophy. Exclusion of celiac disease and wheat allergy diagnosis with improvement of IBS-like symptoms after gluten removal from diet and get worse after gluten reintroduction [62]	Skin biopsy is required for a definitive diagnosis, although antibodies to tissue and epidermal transglutaminase are detectable in the serum of patients [71,72]	There is no test to definitively diagnose IBS [78,79]	Fasting blood glucose level, oral glucose tolerance test, glycosylated hemoglobin supported by serum levels of anti-Islet cell (ICA), anti-insulin (IAA), glutamic acid decarboxylase (GADA) and protein tyrosine phosphatase like protein (IA2) antibodies [99,100,101,102]	Fasting blood glucose level, oral glucose tolerance test, glycosylated haemoglobin [132]
**Comorbidities**	Type 1 diabetes, gastritis and duodenitis, vitamin D deficiency, gastroesophageal reflux disease, thyroiditis, Crohn disease, Down syndrome, Turner syndrome, William’s syndrome, Addison’s disease, vitiligo, psoriasis, iron deficiency, anemia, psychiatric disorders, lymphoma [6]	Psoriasis, fibromyalgia and Chronic Fatigue Syndrome, anemia, headache and psychiatric disorders [56]	Autoimmune thyroid diseases, pernicious anemia, gastric atrophy, type I diabetes, systemic lupus erythematosus, Sjoegren disease, sarcoidosis, vitiligo and alopecia areata, lymphoma [75].	Gastro-esophageal reflux, genito-urinary symptoms, fibromyalgia, headache, backache and psychological symptoms [79,80].	Celiac disease, Grave’s disease, Hashimoto’s disease, Addison disease, vitiligo, autoimmune thyroid disease [98]	Hypertension, Obesity, dyslipidemia, Nonalcoholic Fatty Liver Disease, Sleep apnea [132]
**Pathogenesis**	Autoimmune Disease. Enteropathy caused by dysregulation of both innate and adaptive immune system [12]	Not autoimmune and not allergic disease Enteropathy caused by innate and/or adaptive immune system dysregulation [57,58]	Autoimmune Disease. Specific cutaneous manifestation of celiac disease [73]	Inflammatory bowel disease associated to serotonin dysregulation, bacterial overgrowth and central dysregulation [80]	Autoimmune Disease. Destruction of pancreatic insulin-producing β-cells by an innate and adaptive immune response [98]	Not autoimmune disease caused by Insulin resistance due both to genetic and environmental factors [132]
**Suggested mechanisms of Gluten toxicity**	Induction of adaptive immune response [12,13,14] Increased intestinal permeability [16,17,19] Change in intestinal microbiota [121]	Induction of adaptive immune response [57,58]	Increased intestinal permeability [73,74]	Increased intestinal permeability [94,95] Induction of adaptive immune response [95]	Increased intestinal permeability [122,123] Change in intestinal microbiota [121,123,133] Induction of β-cell iperactivity and dysfunction [130,134] Stimulation of immune system [124,127]	Increased intestinal permeability [122,123] Change in intestinal microbiota [121,123] Induction of β-cell iperactivity and dysfunction [130,135]
